# Earlier cancer diagnosis in primary care: a feasibility economic analysis of ThinkCancer!

**DOI:** 10.3399/BJGPO.2022.0130

**Published:** 2023-02-08

**Authors:** Bethany Fern Anthony, Stefanie Disbeschl, Nia Goulden, Annie Hendry, Julia Hiscock, Zoe Hoare, Jessica Roberts, Jan Rose, Alun Surgey, Nefyn Howard Williams, Daniel Walker, Richard Neal, Clare Wilkinson, Rhiannon Tudor Edwards

**Affiliations:** 1 Centre for Health Economics and Medicines Evaluation (CHEME), Bangor University, Bangor, UK; 2 North Wales Centre for Primary Care Research, Bangor University, Bangor, UK; 3 NWORTH, Bangor University, Bangor, UK; 4 National Cancer Research Institute (NCRI), Consumer Member, London, UK; 5 Department of Primary Care and Mental Health, University of Liverpool, Liverpool, UK; 6 College of Medicine and Health, University of Exeter, Exeter, UK

**Keywords:** primary health care, general practice, feasibility studies, health care economics and organizations, early cancer diagnosis

## Abstract

**Background:**

UK cancer survival rates are much lower compared with other high-income countries. In primary care, there are opportunities for GPs and other healthcare professionals to act more quickly in response to presented symptoms that might represent cancer. ThinkCancer! is a complex behaviour change intervention aimed at primary care practice teams to improve the timely diagnosis of cancer.

**Aim:**

To explore the costs of delivering the ThinkCancer! intervention to expedite cancer diagnosis in primary care.

**Design & setting:**

Feasibility economic analysis using a micro-costing approach, which was undertaken in 19 general practices in Wales, UK.

**Method:**

From an NHS perspective, micro-costing methodology was used to determine whether it was feasible to gather sufficient economic data to cost the ThinkCancer! intervention. Owing to the COVID-19 pandemic, ThinkCancer! was mainly delivered remotely online in a digital format. Budget impact analysis (BIA) and sensitivity analysis were conducted to explore the costs of face-to-face delivery of the ThinkCancer! intervention as intended pre-COVID-19.

**Results:**

The total costs of delivering the ThinkCancer! intervention across 19 general practices in Wales was £25 030, with an average cost per practice of £1317 (standard deviation [SD]: 578.2). Findings from the BIA indicated a total cost of £34 630 for face-to-face delivery.

**Conclusion:**

Data collection methods were successful in gathering sufficient health economics data to cost the ThinkCancer! intervention. Results of this feasibility study will be used to inform a future definitive economic evaluation alongside a pragmatic randomised controlled trial (RCT).

## How this fits in

For many cancers, earlier diagnosis is associated with greater survival and better patient quality of life and experience. Cancer survival is lower in the UK compared with other comparable countries. The need for interventions such as ThinkCancer!, which aims to improve the timely diagnosis of cancer within primary care teams, are increasingly important. Lessons learnt from this feasibility trial have important practice implications to ensure cancer diagnoses are as timely as possible.

## Introduction

Between 2017 and 2018, cancer investigation and treatments accounted for 7.1% of the total NHS expenditure in Wales. This was the fourth highest expenditure category, with a resultant cost of £463 million.^1^ There are also wider societal costs of cancer, such as direct patient costs, including loss of income, travel costs to regular medical appointments, and increased household bills such as heating. Research from Macmillan Cancer Support found that almost one-third of people living with cancer in the UK have experienced a loss of income owing to their cancer diagnosis, with the average income lost at £860 per month.^2^

Previous international studies have shown that Wales is at the bottom of international comparators with respect to cancer survival and have underlined later diagnosis as a leading cause.^1^ If cancer is diagnosed early, treatment is more likely to be curative, less intensive and less costly.^3^ The Welsh Government has outlined a number of challenges with respect to earlier cancer diagnosis, including difficulties among GPs and other healthcare professionals in identifying cancers that present with vague or non-specific symptoms.^3^ A previous study in Wales demonstrated the cost-effectiveness of a rapid diagnosis centre (RDC) for patients presenting with vague or non-specific symptoms of cancer through referral from primary care.^4^ The present study of ThinkCancer! aims to address the gap in diagnostics in primary care and targets the entire general practice team.

The aim of this study was to conduct a feasibility economic analysis of the ThinkCancer! intervention. The ThinkCancer! intervention is a complex behaviour change intervention targeting general practice teams, aiming to improve earlier cancer diagnosis in primary care. Preliminary estimates of cost-effectiveness in feasibility studies with small sample sizes may risk negative outcomes; therefore, the focus should be limited to developing and refining methods for data collection, including health economics data.^5^

The specific objectives of the ThinkCancer! feasibility economic analysis were as follows:

1. to explore the feasibility of gathering sufficient economic data including costs of the intervention to inform a future definitive economic evaluation alongside a RCT;
2. to conduct a BIA and sensitivity analysis to explore the costs of face-to-face delivery of the ThinkCancer! intervention as intended pre-COVID-19.

## Method

**Figure 1.**
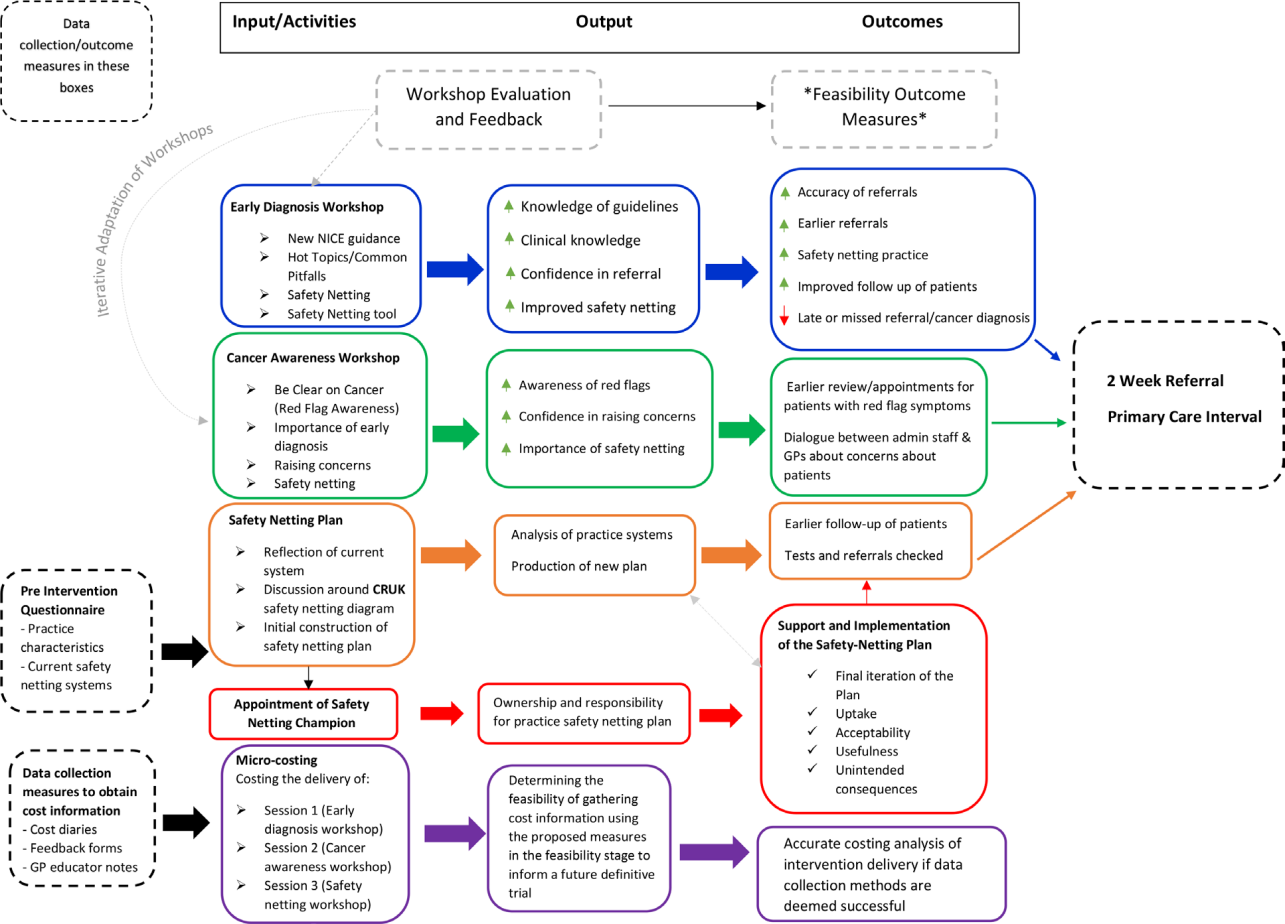
Adapted ThinkCancer! logic model. CRUK = Cancer Research UK. NICE = National Institute for Health and Care Excellence.

From an NHS perspective, a feasibility economic analysis of the ThinkCancer! intervention was conducted. The intervention was carried out in workshops delivered as three separate sessions to each practice individually. The intervention as a whole comprised four main components. The first and second components were educational sessions, one for clinical staff members (the ‘early diagnosis’ session) and the other for patient-facing non-clinical staff members (the ‘cancer aware’ session). The third session included the final two components and was delivered as a safety-netting session to both clinical and non-clinical staff members. The final two components within this session involved the development of a tailored cancer safety-netting plan and the appointment of a cancer safety-netting champion within the practice. A full description of the intervention is published in the study protocol.^6^ The ThinkCancer! logic model presented in the main feasibility protocol^6^ was developed in accordance with the Medical Research Council (MRC) framework for the evaluation of complex interventions.^7^ The logic model depicts the relationship between the intervention components and the intended outcomes for the main feasibility trial. This logic model has been adapted (Figure 1) to demonstrate the point at which costs were collected for the micro-costing analysis presented in this article. The logic model also details the intended output and outcome from the micro-costing activities (Figure 1). Outcome data from the main feasibility study were not included in this feasibility economic analysis.

The intervention was delivered in the following three formats: remote webinars; pre-recorded sessions allowing participants to engage with the materials in their own time; or face-to-face sessions, as originally intended before the COVID-19 pandemic. A micro-costing approach was used to gather sufficient economic data to cost the ThinkCancer! intervention. In micro-costing, each component of resource use is estimated and then combined with its unit cost to allow accurate costing of the intervention.^8,9^

Data on the direct medical costs of the intervention were collected using health economics cost diaries (Supplementary File 1) completed by those delivering the intervention. Costs recorded in the cost diaries included intervention deliverer costs and material costs. It was intended to use workshop feedback forms to cost NHS staff time attending the intervention (Supplementary File 2). However, early on during the study it became apparent that it was unfeasible to use the feedback forms to obtain this data owing to incorrect information provided by staff members regarding their attendance at the sessions. Consequently, the data obtained in the feedback forms were not used. Instead, the notes written by the GP educator delivering the workshop were to determine the numbers and roles of each staff member in attendance at each of the sessions.

Staff time participating in the workshops was costed using Personal Social Services Research Unit (PSSRU) national unit costs.^10^ Unit costs for non-clinical staff were calculated using the NHS Agenda for Change annual salaries^11^ to derive a cost per hour, as unit costs for non-clinical primary care staff were not available in the PSSRU. A sample unit costs schedule for primary care practice staff costs can be viewed in Table 1. Relevant overheads and on-costs were included in the calculations. As part of their methodology, overheads and on-costs were accounted for in the unit costs provided by the PSSRU.^10^ Where no on-cost information was available, a flat rate of 30% was applied, based on estimates used in the PSSRU.^10^ To avoid double counting, overheads were not applied when costing non-clinical staff as this was assumed to fall within the administration and management support overheads included within the PSSRU clinical staff unit costs.^10^ The upfront costs of developing the intervention to be delivered in an online format (live and pre-recorded sessions) were assessed, and means and descriptive statistics were produced relating to the costs of delivering the intervention across different practices. The follow-up period for outcome measures in the main feasibility study was 6 months, this economic analysis did not include any outcome data; therefore, in accordance with National Institute for Health and Clinical Excellence (NICE) guidelines a discount rate was not applied as the costs and benefits were not beyond 12 months.^12^

**Table 1.** Total costs for intervention delivery at 19 general practices

Type of cost	Total	Mean	SD
Materials	£3149	£166	52.88
Staff attendee time	£18 773	£988	524.95
Intervention deliverer time	£3109	£164	53.37
	£25 030	£1317	578.24

The base-case analysis explored the cost of ThinkCancer! as it was delivered to 19 general practices in Wales. The analysis presents total costs of intervention delivery, which refers to the costs for each of the participating practices to receive one multi-component workshop, which consisted of three separate workshops or sessions. Each multi-component workshop was delivered to individual practices separately; that is, one intervention (delivered as three separate workshops or sessions to each of the 19 practices). Upfront intervention development costs are presented separately and do not come under total costs of intervention delivery. Recruitment of practices occurred in the following two stages: one practice was recruited before the COVID-19 pandemic and then the study was put on hold. Recruitment then resumed in July 2020. The total study recruitment period was between February 2020 and February 2021, and intervention delivery ran from October 2020 to May 2021. Health economics data to cost intervention delivery was collected immediately following each workshop. The majority of workshops were delivered to practices remotely in an online live delivery format; however, using BIA and sensitivity analysis, it was also explored what the costs would be of delivering the intervention face to face as intended pre-COVID-19. BIAs are economic assessments used to explore the financial consequences of implementing a new health technology or intervention and are used to aid in decision making with respect to the allocation and reallocation of resources within specific healthcare settings with finite resources.^13,14^ A sensitivity analysis was conducted to show the difference in costs if the intervention was delivered face to face by the GP educator alone, and with the addition of one support role assisting the intervention. A separate BIA was also conducted using published statistics on the number of active general practices in Wales during the time of this study and the findings of the base-case analysis to project the cost of delivering ThinkCancer! remotely at scale to practices across Wales.

In a separate BIA, the cost of delivering ThinkCancer! remotely at scale to general practices across Wales was explored, based on the average intervention delivery cost per practice from the base-case analysis. According to Welsh Government statistics,^15^ there were 404 active general practices across Wales in 2020. Based on an average cost per practice of £1317, the estimated total cost of delivering ThinkCancer! online to all general practices across Wales would be approximately £532 000.

## Results

### Feasibility of gathering sufficient health economics data to cost ThinkCancer!

Cost diaries developed for this feasibility trial successfully captured all of the direct medical costs of the intervention, with no missing data. As discussed earlier, feedback forms were not used owing to inaccurate information being provided. As each workshop was split into three sessions, some staff members attended one of the first two sessions and had then completed the feedback forms with the intention of attending the third session (safety-netting session), but then had subsequently not attended. Inaccuracies in the number of staff attending sessions became apparent when the information in the feedback form did not correspond with the GP educator notes on attendance. Consequently, the analysis did not use any information captured in the feedback forms and, instead, GP educator notes were used to determine the number and roles of staff attending the sessions. Nevertheless, this sometimes proved difficult owing to the online delivery format that was primarily used to deliver the intervention. In some instances, it was difficult to ascertain the number and roles of staff attending the workshops owing to factors such as cameras being switched off during the workshops and more than one member of staff watching the workshops over one computer screen. It was also inappropriate and unfeasible to gather information on staff pay bands owing to the online mode of delivery adopted; therefore, average costs of each staff role were used.

### Base-case analysis

The total cost of delivering the ThinkCancer! intervention to the 19 practices was £25 030. This was the total cost for each practice to receive one full workshop (split into three sessions). This cost comprised of materials (including postage costs), staff attendee time, and intervention delivery time (Table 1). Nineteen multi-component workshops (each split into three sessions) were delivered in total (one to each practice). Of these, 15 practices received all three of their sessions in a live online delivery format. Three practices out of the 19 practices, received one of their three sessions in a pre-recorded format and the remaining two sessions in a live online format. One practice out of the 19 practices received two of their three sessions in an online live format but received the third session in a face-to-face format; travel costs were therefore also calculated in this instance.

The mean cost of intervention delivery per practice was £1317 (SD 578.24, range £431–£2498). This was calculated by dividing the total cost of intervention delivery across the 19 practices by 19 (the number of practices that received the intervention). The largest driver of cost was staff attendance at the workshops. The total cost of primary care staff time for attending the intervention for all 19 general practices was £18 773 (Table 1). GP attendance yielded the highest cost, at a unit cost of £2.60 per minute (Supplementary File 3).

Total cost of materials across the practices (including postage costs) was £3149 (Table 1). The highest costing item was the Red Whale handbook (£21.54 each, including value-added tax) (Table 2). The total time to deliver the intervention was 37.55 hours (2253 minutes). Table 3 provides a breakdown of the costs of GP educator and support role time to deliver the intervention. The total cost of intervention deliverer time at the 19 general practices was £3109. Separate upfront intervention development costs were estimated to be £4385 based on 87 hours of GP educator time to construct and develop the ThinkCancer! intervention.

**Table 2.** Total costs of materials for ThinkCancer! intervention delivery at 19 general practices

Materials	Units	Unit cost (£)	Number of units	Total cost (£)
Red Whale handbook	One Red Whale handbook (with value-added tax)	£21.54	114	£2455.56
SSNAP tool	Per tool	£1.78	72	£128.16
Feedback form	Per form	£0.15	280	£42
Participant information sheet	Per sheet	£0.50	570	£285
Consent form	Per form	£0.10	258	£25.80
Cancer awareness pack	Per pack	£1.65	19	£31.35
Postage	NA	NA	Postage of materials to 19 practices	£180.71
				£3148.58

SSNAP = Shared Safety Net Action Plan.

**Table 3.** Total costs of time to deliver ThinkCancer! at 19 practices

Intervention deliverer	Unit	Unit costs	Total time of intervention deliveryacross 19 practices (minutes)	Travel costs(£0.40 per mile)	Total cost (£)
GP educator	Per minute	£0.84	2253	£41.60	£1934.12
Support role 1	Per minute	£0.34	2193	£0	£745.62
Support role 2	Per minute	£0.48	894	£0	£429.12
					£3108.86

### Budget impact and sensitivity analysis

Only one practice received one of their workshop sessions in a face-to-face format. A BIA was conducted to explore what the total costs would be for face-to-face delivery of the ThinkCancer! intervention if all of the 19 general practices recruited in this feasibility study received all of their workshop sessions in a face-to-face format. Table 4 provides a breakdown of additional costs incurred for face-to-face delivery including travel expenses (calculated at £0.40 per mile) and travel time duration (total minutes for return journey to each practice) in order to calculate intervention deliverer time costs for travel. Distances and travel durations between the practices and the research centre were obtained from Google Maps.

**Table 4.** Estimated travel expenses and travel-time costs for intervention deliverer for face-to-face delivery format at 19 general practices

Total miles for return journeys to the general practice sites	Total cost for travel expenses (calculated at £0.40 per mile)	Total travel time to 19 general practices (minutes)	Total cost of intervention deliverers’ time for travel (based on the number of deliverers at workshops in the base-case analysis)	Total cost of face-to-face delivery
3733	£1493.20	5606	£8107	£34 630

Estimated total costs for face-to face delivery at the 19 general practices would be £34 630 based on the total costs accrued for intervention delivery in the base-case analysis and additional costs for travel expenses (one vehicle shared by the intervention deliverers) and intervention deliverer travel time (Table 4). Estimated total costs for travel expenses were £1493 (calculated at £0.40 per mile). Total costs of intervention deliverer travel time was £8107. The number of intervention deliverers in attendance varied between practices but usually there were two roles delivering and/or supporting the intervention; however, an additional support role was in attendance for some of the workshops. Sensitivity analysis shows that the total costs of face-to-face delivery by one GP educator (including travel expenses for one vehicle and GP educator travel time) was estimated at £31 232. With the addition of one support role, the total costs for face-to-face delivery (including travel expenses for one shared vehicle and travel time costs) is estimated at £33 138.

In a separate BIA, the cost of delivering ThinkCancer! remotely at scale to general practices across Wales was explored, based on the average intervention delivery cost per practice from the base-case analysis. According to the Welsh Government,^15^ there were 404 active general practices across Wales in 2020. Based on an average cost per practice of £1317, the estimated total cost of delivering ThinkCancer! online to all general practices across Wales would be approximately £532 068.

## Discussion

### Summary

The total costs of delivering the ThinkCancer! intervention across 19 general practices in Wales was £25 030. Costs per practice ranged from £431–£2498, with an average cost per practice of £1317 (SD 578.24). The potential budget impact if the intervention were to be delivered face to face would be £34 630. Sensitivity analysis revealed that if the intervention were to be delivered by one GP educator, the total estimated cost for face-to-face delivery would be £31 232. With the addition of one support role assisting the GP educator with the intervention delivery, the total cost of face-to-face delivery is estimated to be approximately £33 138.

### Strengths and limitations

This feasibility economic analysis provides estimated costs of delivering the ThinkCancer! intervention in primary care settings. ThinkCancer! is a relatively low-cost intervention when compared with the costs associated with later stage diagnosis. For example, treatment costs per patient for early stage ovarian cancer is £5328 but increases to £15 081 at its latest stage.^16^ This intervention has the potential to expedite the diagnosis of symptomatic cancer, improve cancer patient outcomes, and avoid costs to the NHS associated with later stage cancer treatments.

Only one of the practices received part of their workshop (one session) in a face-to-face format making it difficult to draw conclusions regarding the feasibility of this mode of delivery. As this was a feasibility study, a cost-effectiveness analysis was not undertaken as it was unlikely that a difference in the primary outcome measure would have been observed owing to the small sample size and short follow-up. Nevertheless, the purpose of this feasibility study was to assess whether it was possible to conduct the proposed intervention in the planned way in order to design a future definitive trial.^5^ Preliminary estimates of cost-effectiveness in feasibility studies with small sample sizes may risk negative outcomes; therefore, the focus of health economics in feasibility studies should be limited to developing and refining methods for data collection, including health economics data.^5,17^ The cost diaries designed for this feasibility stage were a successful method of gathering sufficient economic data to cost the ThinkCancer! intervention. However, capturing information on the number and roles of primary care staff who attended the intervention was challenging owing to the online delivery format. Consequently, it is possible that some costs may have been underestimated in the analysis as there is uncertainty on the exact numbers of staff who received the intervention remotely. These issues will be discussed and reviewed with the ThinkCancer! research team when planning and designing a future definitive trial.

### Comparison with existing literature

There is a lack of evidence relating to the costs of interventions to expedite cancer diagnosis in general practice settings. One UK study from 2020 assessed the cost-effectiveness of a RDC for patients presenting with vague non-specific symptoms suspicious of cancer through referral from primary care. The results found that the RDC yielded more quality-adjusted life years (QALYs) and was less costly than standard clinical practice when the RDC was running at a capacity of ≥80%.^4^ To the authors' knowledge, the ThinkCancer! study is the first costing study of an intervention to expedite cancer diagnosis within general practice settings that targets the entire general practice team.

A systematic review and meta-analysis of methods for training licensed healthcare professionals to deliver clinical interventions concluded that future trials should explore the cost-effectiveness of online versus alternative methods of training.^18^ Owing to the COVID-19 pandemic, the ThinkCancer! intervention was mostly delivered in a digital format. Previous studies have explored the cost-effectiveness of e-learning interventions and online continued professional development (CPD) for clinicians in the areas of falls prevention education,^19^ suicide prevention,^20^ and online cancer education for nurses and allied health professionals.^21^ To the best of the authors' knowledge, the present study is the first of its kind to explore the costs of an online intervention aimed at improving the diagnosis of cancer in primary care, and consequently adds value to the current evidence base in this area, especially since the increased use of online delivery formats since the COVID-19 pandemic.

### Implications for research and practice

Results from this feasibility study will be used to inform a future definitive economic evaluation alongside a pragmatic RCT. The costings from this feasibility economic analysis may be helpful for future economic modelling studies. Moreover, the findings from this study may be useful for researchers who are setting up economic evaluation models of training for earlier diagnosis in other clinical areas such as stroke in primary care.

There has been a large impact from the COVID-19 pandemic on cancer diagnosis and treatment.^22^ Primary care interventions that expedite the diagnosis of symptomatic cancer have the potential to reduce large costs to the NHS and improve patient and carer outcomes; as later stage cancer treatments are often longer, more aggressive to patients, with larger associated healthcare costs, compared with earlier stage treatment. The ThinkCancer! intervention has the potential to expedite the diagnosis of symptomatic cancer, improve cancer patient outcomes, and may avoid costs to the NHS associated with later stage cancer treatments. Lessons learnt from this feasibility trial have important practice implications to ensure cancer diagnoses are as timely as possible.

## Trial registration number

ClinicalTrials.gov NCT04823559.

## Data

The dataset relied on in this article is available from the corresponding author on reasonable request.
